# Convolutional neural networks for detection of transthyretin amyloidosis in 2D scintigraphy images

**DOI:** 10.1186/s13550-022-00897-9

**Published:** 2022-05-07

**Authors:** Hanna-Leena Halme, Toni Ihalainen, Olli Suomalainen, Antti Loimaala, Sorjo Mätzke, Valtteri Uusitalo, Outi Sipilä, Eero Hippeläinen

**Affiliations:** 1 Clinical Physiology and Nuclear Medicine, Helsinki University Hospital and University of Helsinki, Helsinki, Finland; 2 Heart and Lung Center, Helsinki University Hospital and University of Helsinki, Helsinki, Finland; 3 Research Program in Systems Oncology, Faculty of Medicine, University of Helsinki, Helsinki, Finland; 4 Department of Physics, University of Helsinki, Helsinki, Finland

**Keywords:** Amyloidosis, Transthyretin, Scintigraphy, Deep learning, Convolutional neural network

## Abstract

**Background:**

Transthyretin amyloidosis (ATTR) is a progressive disease which can be diagnosed non-invasively using bone avid [^99m^Tc]-labeled radiotracers. Thus, ATTR is also an occasional incidental finding on bone scintigraphy. In this study, we trained convolutional neural networks (CNN) to automatically detect and classify ATTR from scintigraphy images. The study population consisted of 1334 patients who underwent [^99m^Tc]-labeled hydroxymethylene diphosphonate (HMDP) scintigraphy and were visually graded using Perugini grades (grades 0–3). A total of 47 patients had visual grade ≥ 2 which was considered positive for ATTR. Two custom-made CNN architectures were trained to discriminate between the four Perugini grades of cardiac uptake. The classification performance was compared to four state-of-the-art CNN models.

**Results:**

Our CNN models performed better than, or equally well as, the state-of-the-art models in detection and classification of cardiac uptake. Both models achieved area under the curve (AUC) ≥ 0.85 in the four-class Perugini grade classification. Accuracy was good in detection of negative vs. positive ATTR patients (grade < 2 vs grade ≥ 2, AUC > 0.88) and high-grade cardiac uptake vs. other patients (grade < 3 vs. grade 3, AUC = 0.94). Maximum activation maps demonstrated that the automated deep learning models were focused on detecting the myocardium and not extracardiac features.

**Conclusion:**

Automated convolutional neural networks can accurately detect and classify different grades of cardiac uptake on bone scintigraphy. The CNN models are focused on clinically relevant image features. Automated screening of bone scintigraphy images using CNN could improve the early diagnosis of ATTR.

## Background

Cardiac amyloidosis is a progressive disease characterized by myocardial deposition of misfolded proteins, mainly transthyretin or light chain amyloid fibrils, which eventually leads to progressive heart failure [1]. Cardiac amyloidosis can be diagnosed by either invasive biopsy or non-invasively by cardiac multimodality imaging [1].

Non-invasive diagnosis of transthyretin amyloidosis (ATTR) is based on nuclear imaging using bone avid [99mTc]-labeled radiotracers such as pyrophosphate (PYP), diphosphono-1,2-propanodicarboxylicacid (DPD) and hydroxymethylene diphosphonate (HMDP) [1]. Thus, ATTR is also an occasional incidental finding on standard bone scintigraphy imaged due to non-cardiac reasons, which is also associated with increased mortality [2]. Currently, novel amyloid-stabilizing treatments are entering clinical practice, and early detection of ATTR has become a topic of interest, as the efficacy of medical interventions is greatest at the early stages of the amyloid cardiomyopathy [3]. Unfortunately, the diagnostic delay of ATTR is commonly long and the patients often have poor quality of life due to advanced amyloid cardiomyopathy at the time of the diagnosis [4].

Nuclear medicine departments could play an important role in the early diagnosis of ATTR by screening cardiac uptake on all bone scintigraphy images. The related workload could be reduced by an automated deep learning-based workflow. In addition, automated image analysis could detect unnoticed cardiac uptake or retrospectively screen large databases of scintigraphy images, which could lead to earlier detection and improved prognosis of ATTR. This would also facilitate novel preventive clinical studies for patients at the early stages of their disease process. Moreover, automated detection of cardiac uptake could notify the technician performing the study to obtain additional cross-sectional imaging of the heart to identify the radiotracer accumulation specifically in the myocardium, and not in the blood pool [1].

Convolutional neural networks (CNN) have proven to be efficient particularly in the classification and segmentation of 2D images [5–10]. Cardiac amyloidosis has been detected with CNN from cardiac magnetic resonance and PET images [11, 12]. However, to our knowledge, CNNs have not been applied in the detection of ATTR on bone scintigraphy. In this study, we apply CNN models to 2D bone scintigraphy for automated detection and classification of patients with ATTR. We present two CNN architectures and compare their classification performance to four state-of-the-art CNNs designed for 2D image classification. Our aim is to use CNNs to automatically detect clinically significant cardiac uptake from bone scintigraphy images.

## Methods

### Patients and imaging protocol

The study population consists of 1334 patients who underwent bone scintigraphy between 2012–2021 in four Finnish nuclear medicine units. The data were collected using standard clinical single-photon emission computed tomography (SPECT) scanners: Philips/ADAC Forte (Philips Healthcare, Eindhoven, The Netherlands; *N* = 100), Philips Brightview (*N* = 200), Siemens e.cam (Siemens Healthcare, Erlangen, Germany; *N* = 547), Siemens Symbia (*N* = 372), GE Infinia Hawkeye (GE Healthcare, Waukesha, Wisconsin, USA; *N* = 96), and GE Discovery 670 (*N* = 19). Low-energy high-resolution (LEHR) collimators were used in all scanners.

Of all participants, 1319 were scanned using a whole-body bone scintigraphy protocol, and 15 patients with thoracic planar scintigraphy included in the clinical cardiac amyloidosis imaging protocol. The emphasis of patient selection was in the inclusion of patients with positive cardiac uptake as their prevalence is low in overall population. All studies were performed using [99mTc]Tc-HMDP imaged at three-hours post-injection. The administered activity was 500–700 MBq. Both visual and CNN analysis of bone scintigraphy data were done for research purposes only. The study was approved by the ethics committee of Helsinki University Hospital and was conducted according to the Declaration of Helsinki.

### Visual analysis of cardiac uptake

**Fig. 1:**
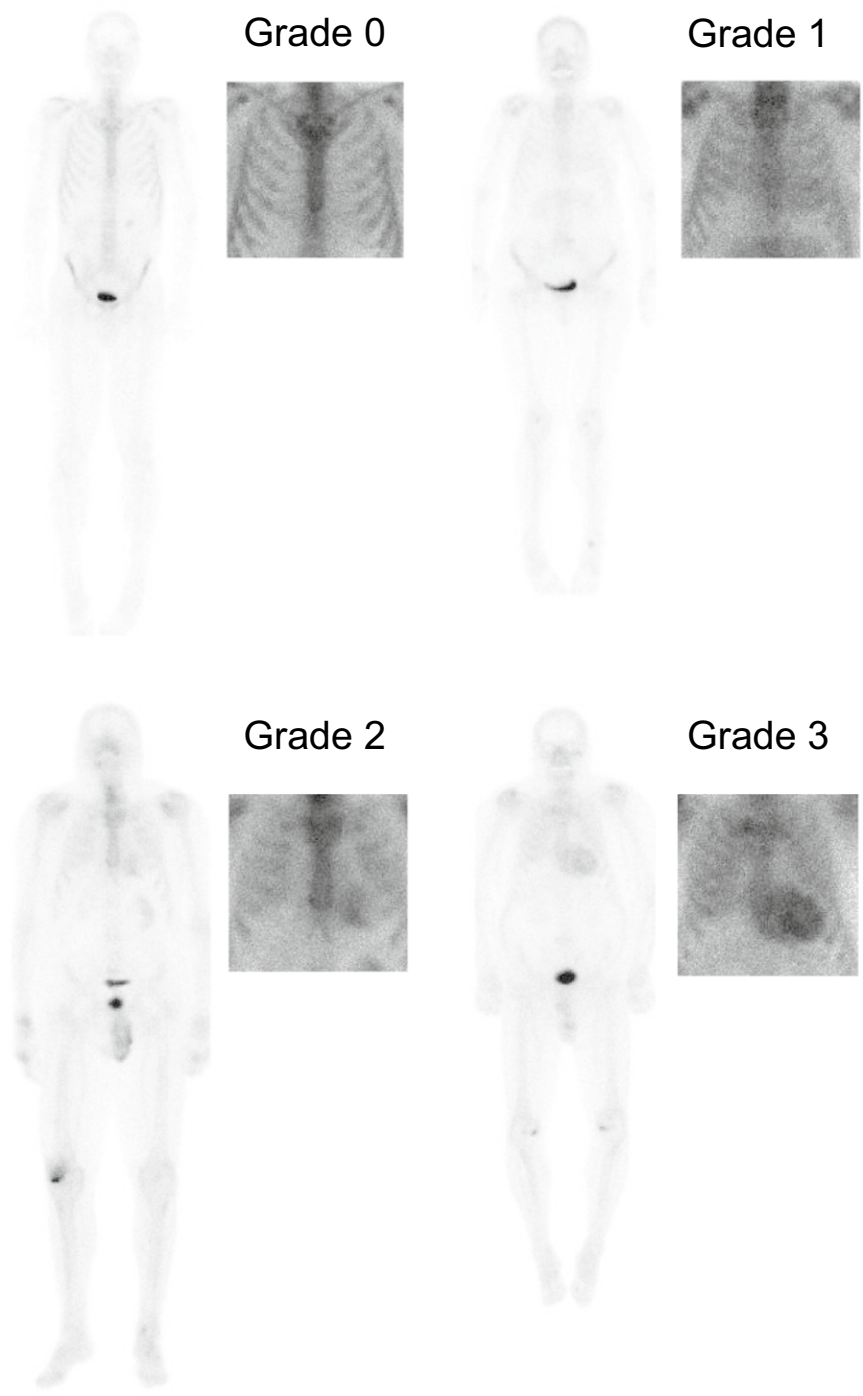
Whole-body and cropped bone scintigraphy images of patients with different Perugini grades of cardiac uptake (0–3)

Three physicians participated in grading bone scintigraphy images for cardiac uptake. All patients with a positive scan (≥ grade 2) were reviewed by one nuclear medicine physician with most clinical experience in amyloid imaging. Different grades of cardiac uptake are demonstrated in Fig. 1. The figure shows both the original whole-body images and the corresponding preprocessed and cropped images used in our further analyses.

The grading of cardiac uptake was visually defined from images using the standard Perugini grade for cardiac uptake [1, 13]:

- Grade 0: no cardiac uptake
- Grade 1: cardiac uptake less than bone uptake
- Grade 2: cardiac uptake with intensity similar to bone uptake
- Grade 3: cardiac uptake greater than bone uptake

Cases borderline for positive (grade 1–2) were graded as positive to optimize the sensitivity of our automated image analysis. We analyzed the reproducibility of the visual grading using 40 anonymized patients. Two nuclear medicine physicians (VU and SM) graded the patients twice. The patients were presented in randomized order, and the physicians were unaware of the prevalence of each Perugini grade in the dataset. Both intra- and interobserver reliability were evaluated using Cohen’s kappa coefficient [14].

### Data preprocessing

**Fig. 2:**
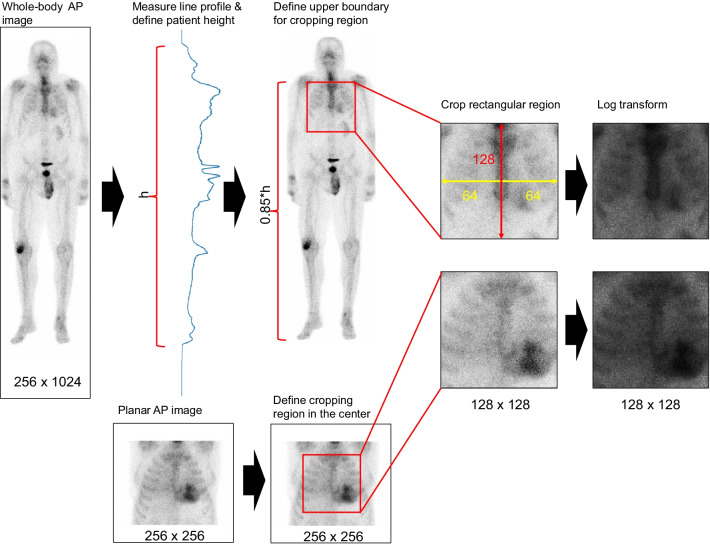
Preprocessing workflow for whole-body and thoracic planar images

Only anterior (AP) images were retained for further analyses since the inclusion of posterior (PA) images did not improve classification accuracy in preliminary tests. The preprocessing workflow is illustrated in Fig. 2. Images were cropped into a 128 × 128 matrix centered at the thoracic region using an automated Python workflow. The location of the cropping region in whole-body images was determined as follows: first, we measured a line profile in the y-direction and found all nonzero pixels, corresponding to the position of the patient in the image. Next, the upper edge of the cropped image was positioned at a height corresponding to 0.85 × patient height. The lower edge was set 128 pixels lower than the upper edge. The left and right edges were set 64 pixels to the left and right from the image center, respectively.

In case of planar images, the image matrix was down sampled to 256 × 256 resolution if necessary, and a 128 × 128 region was cropped at the center of the 256 × 256 matrix.

Finally, all nonzero pixel intensities in both cropped whole-body and planar images were log-transformed in order to reduce the intensity of possible hot spots (e.g., injection site and uptake in bones due to injury, small fracture or metastatic lesion), which could negatively affect the classification results. The data were anonymized during preprocessing, so that the data used in further analyses included only the cropped image matrix, patient’s age and Perugini grade.

### CNN models

We developed two CNN models, referred to as Linear and Residual. The models were implemented using Python 3.8.3 and functions in Tensorflow 2.2.0 [15] and Keras 2.3.0 [16]. The Linear model included one convolutional layer, followed by four convolution blocks with six consecutive convolutional layers (3 × 3 kernel, stride 1, ReLu activation function) and one average pooling layer (2 × 2 kernel, stride 1) in each. After the convolution blocks, there was a flattening layer, a dropout layer (dropout 0.2) and the final fully connected layer (softmax activation function, dimension equal to the number of classes).

**Fig. 3:**
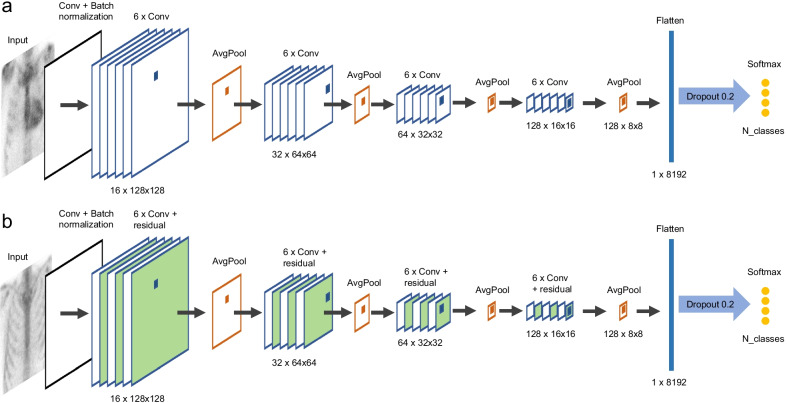
Architectures for Linear **a** and Residual **b** models. In **b**, convolutional layers including skip connections are shown in green. The dimensionality of each layer is shown below the layers. N_classes refers to the number of classes, which was either 4 or 2 depending on the classification task

The Residual model was otherwise similar to the Linear model but included skip connections between every other convolutional layer, i.e., the weights of the layer n–2 were added to layer *n* and the activation function was applied to this summed layer (see details in [17]). The architectures of these models are shown in Fig. 3.

For comparison, we classified data with four state-of-the-art models implemented in Keras library: VGG16 [18], ResNet50 [17], InceptionV3 [19] and MobileNet [20]. For all these models, we used the pre-trained versions with ImageNet weights as initial weights. The output layer of all state-of-the-art models was omitted and replaced with similar flattening layer, dropout layer and fully connected layer as implemented in the Linear and Residual models.

### CNN training and validation

We first classified the images using the visually determined Perugini grades (0, 1, 2 and 3) as the ground truth labels (four-class classification). We also studied the accuracy of CNN for detection of positive (grade > 2) and negative (grade < 2) cardiac uptake for ATTR and differentiation of high-grade (grade 3) cardiac uptake from other patients.

Similar training and validation procedures were used for all CNN models. We quantified the classification accuracy with fivefold cross-validation, in which 80% of the data were used for training and 20% for testing the CNNs. We used stratified cross-validation, i.e., the proportion of different classes in each cross-validation fold was equal.

As the size of the dataset was limited, we increased the number of training images by data augmentation. On each cross-validation fold, 5330 augmented images were generated by shifting (range ± 10% in both x- and y-directions), rotating (range ± 20°), and scaling (range ± 20%) the original training images in a randomized way, using the *ImageDataGenerator* function implemented in Keras. Finally, both the training and testing images were z-score normalized pixel-wise with respect to the training data, i.e., the average of the training images was subtracted from each image and subsequently, the image was divided with the standard deviation of the training images.

The model training process was carried out with 50 epochs and a batch size of 128. As the dataset was imbalanced, i.e., the number of patients with grades 0 and 1 was significantly higher than that of patients with grades 2 and 3, we decided to use class weights in the classification. The penalty for misclassification of the minority classes (grades 2 and 3) was set higher than that of the majority classes (grades 0 and 1) in order to overcome the uneven distribution in the dataset. For model optimization, we used sparse categorical cross-entropy loss function and an Adam optimizer with an initial learning rate 1e–4. Ten percent of the training data were used for validation during the training process. The validation data were used for guiding the training, so that the learning rate was reduced whenever the validation loss did not decrease in two consecutive epochs; the minimum learning rate was set to 1e–7. Classification performance was evaluated by receiver operating characteristic (ROC) analysis. We calculated the area under the curve (AUC), total accuracy and class-specific precision (number of true positives over the number of true positives plus the number of false positives) and recall (number of true positives over the number of true positives plus the number of false negatives) for each CNN model using the roc_auc_score function implemented in Scikit-Learn [21]. The above training and testing process took about 1 h on an NVIDIA Quadro P5000 graphical processing unit.

After classifying all patients, we investigated whether bone metastases have an effect on the classification results of the CNN. We selected only the Residual CNN for this analysis for simplicity. As we had the final CNN classification for each patient, we divided them to those with and without bone metastases. Thereafter, AUC and total accuracy were calculated separately for these two groups.

### CNN layer visualization

Besides detection and classification of cardiac uptake on bone scintigraphy, we studied which parts of the image contribute most to the CNN output, i.e., “what the CNN is looking for”. We visualized the maximum activation maps of layers 2, 10, 17, and 24 of the Linear model and layers 2, 12, 22, and 32 of Residual model, corresponding to the first convolutional layer of each convolution block.

## Results

### Patient characteristics

The study involved 1334 patients of which 245 were females (18%) with mean age of 77 ± 10 years. The patients were imaged due to prostate cancer (*N* = 1013), breast cancer (*N* = 159), other cancer (*N* = 81), orthopedic or metabolic indication (*N* = 55) or with suspicion of cardiac amyloidosis (*N* = 26). Grade 1 cardiac uptake was found in 296 patients (22.2%), grade 2 in 23 patients (1.7%) and grade 3 in 24 patients (1.8%) [2]. A positive study (grade ≥ 2 cardiac uptake) was found in 31 patients with prostate cancer, 3 patients with breast cancer, 2 patients with other cancer and 11 patients in whom ATTR was suspected.

### Intra- and interobserver reliability

Cohen’s kappa for detection of significant cardiac uptake (≥ grade 2) was very good for the intraobserver variability (0.89) and good for interobserver variability (0.79). Similarly, Cohen’s kappa for both intra- and interobserver variability of different Perugini grades was good (0.79 and 0.69, respectively).

### Four-class Perugini grade classification of cardiac uptake

In four-class classification of Perugini grade, VGG16 obtained the best total accuracy (0.74) and AUC (0.87). Our custom-made models obtained nearly similar AUCs: 0.86 for both Linear and Residual model. VGG16 yielded better total accuracy mainly due to a better recall for grade 0; however, it performed worse than our models in detection of classes 1, 2 and 3.

Grade 3 uptake got the highest classification scores with both Linear (precision 0.91, recall 0.83) and Residual (precision 0.83, recall 0.83) models.

Grades 1 and 2 were the most difficult classes to discriminate. The Residual model yielded the best recall for both grade 1 (0.65), and grade 2 (0.65).

Grade 0 was well classified with all models, but the best precision (0.88) was obtained with the Residual model. As the majority (73%) of the patients in our dataset represented grade 0, it was crucial that the other grades were not misclassified as grade 0, i.e., high precision for this class was preferred over high recall. On the contrary, as grade 3 represented only 1.7% and grade 2 1.8% of the data, it was important that these rare cases were classified with a high recall, i.e., few false negatives. The Linear and Residual models both yielded the highest precision for grade 0 and highest recall for grades 2 and 3.

The cross-validated results for four-class classification are summarized in Table 1.

**Table 1:** Cross-validated results for automated CNN four-class Perugini grade classification

	AUC	ACC	Grade 0		Grade 1		Grade 2		Grade 3	
			Precis	Recall	Precis	Recall	Precis	Recall	Precis	Recall
Linear	0.86	0.66	0.86	0.68	0.37	0.59	0.29	0.61	0.91	0.83
Residual	0,86	0,67	0,88	0,68	0,40	0,65	0,22	0,65	0,83	0,83
VGG16	0.87	0.74	0.83	0.83	0.44	0.45	0.46	0.57	1.00	0.79
ResNet50	0.78	0.66	0.81	0.78	0.33	0.31	0.11	0.04	0.20	0.76
InceptionV3	0.75	0.62	0.80	0.70	0.30	0.40	0.06	0.13	0.59	0.64
MobileNet	0.76	0.74	0.76	0.97	0.42	0.07	0.00	0.00	1.00	0.17

ACC = accuracy; AUC = area under the curve; Precis = precision

### Detection of high-grade cardiac uptake

Differentiation of severe grade 3 cardiac uptake from other patients yielded better results than four-class Perugini grade classification. Grades 0–2 were classified with precision and recall of 0.99–1 with all models. For grade 3, the best precision and recall were achieved with the Residual model (0.83 and 0.89, respectively). Also in this case, Keras’ pre-trained models did not perform as well as our custom-made CNN models in the classification of grade 3. The results are shown in Table 2.

**Table 2:** Cross-validated results for classification of Perugini grade 0–2 vs grade 3 cardiac uptake

	AUC	Accuracy	Grade 0–2		Grade 3	
			Precision	Recall	Precision	Recall
Linear	0.94	0.99	1.00	0.99	0.73	0.89
Residual	0.94	0.99	1.00	1.00	0.83	0.89
VGG16	0.92	0.99	1.00	0.99	0.62	0.85
ResNet50	0.82	0.99	0.99	1.00	0.94	0.63
InceptionV3	0.80	0.99	0.99	1.00	0.80	0.59
MobileNet	0.72	0.99	0.99	1.00	0.92	0.44

### Automated detection of significant cardiac uptake

Classification of negative (grade < 2) and positive (grade ≥ 2) cases of cardiac uptake was more challenging than discriminating high-grade (grade 3) cardiac uptake from other patients. The best AUCs were achieved using the Residual (0.90) and Linear (0.88) models. VGG16 yielded the best total accuracy (0.98). For detection of negative patients, all models performed reasonably well in terms of precision and recall, which were both > 0.95 with all models except ResNet50 which resulted in a recall of 0.80. For detection of positive ATTR patients, ResNet50 achieved the best recall (0.90), but the poorest precision (0.14). The best precision (0.81) was obtained with VGG16; however, the recall for that model was only 0.54. The Linear model achieved both high precision (0.59) and recall (0.79). Also, the Residual model yielded a high recall (0.85) as shown in Table 3.

**Table 3:** Cross-validated results for automated detection of patients with positive cardiac uptake suggestive for ATTR

	AUC	Accuracy	Negative *patients*		Positive patients*	
			Precision	Recall	Precision	Recall
Linear	0.88	0.97	0.99	0.98	0.59	0.79
Residual	0,89	0,97	0,99	0,98	0,58	0,81
VGG16	0.77	0.98	0.98	1.00	0.81	0.54
ResNet50	0.85	0.80	1.00	0.80	0.14	0.90
InceptionV3	0.78	0.95	0.98	0.97	0.39	0.60
MobileNet	0.76	0.95	0.98	0.96	0.35	0.56

^*^Perugini grade ≥ 2

### The effect of bone metastases on automatic image analysis

The presence of bone metastasis did not significantly affect the automatic Perugini grading or detection of positive cardiac uptake. The accuracy in four-class Perugini grade classification was 0.71 for patients with and 0.66 for those without metastases and AUCs were 0.43 and 0.51, respectively. In classification of positive vs. negative study, the results were similar regardless of the presence of metastases. Total accuracies were 0.97 and 0.98 for patients with and without metastases and AUCs were 0.90 and 0.89, respectively.

### CNN layer visualization

**Fig. 4:**
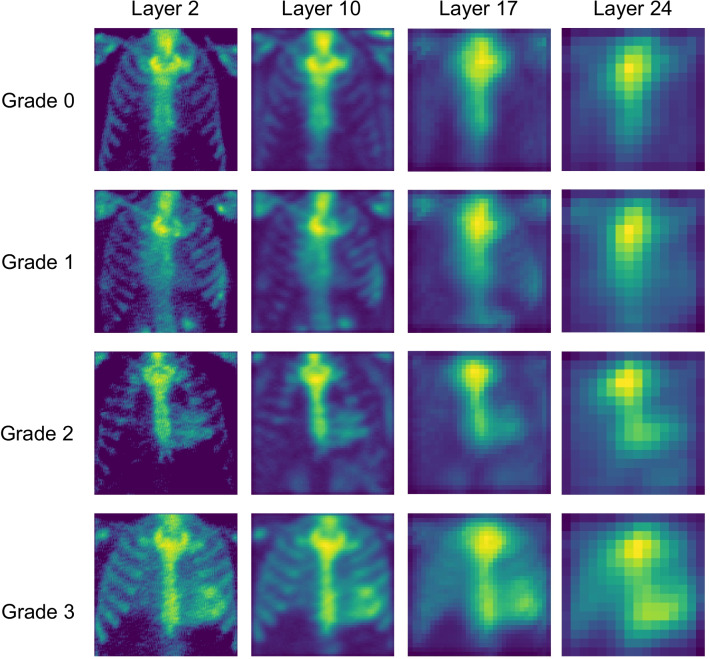
Maximum activation maps for layers 2, 10, 17 and 24 of the Linear model for input images representing different grades of cardiac uptake. Activation maps demonstrate that the convolutional neural network model finds abnormal myocardial signal in patients with cardiac uptake suggestive for ATTR and not extracardiac features, similarly to standard clinical reading by a physician

**Fig. 5:**
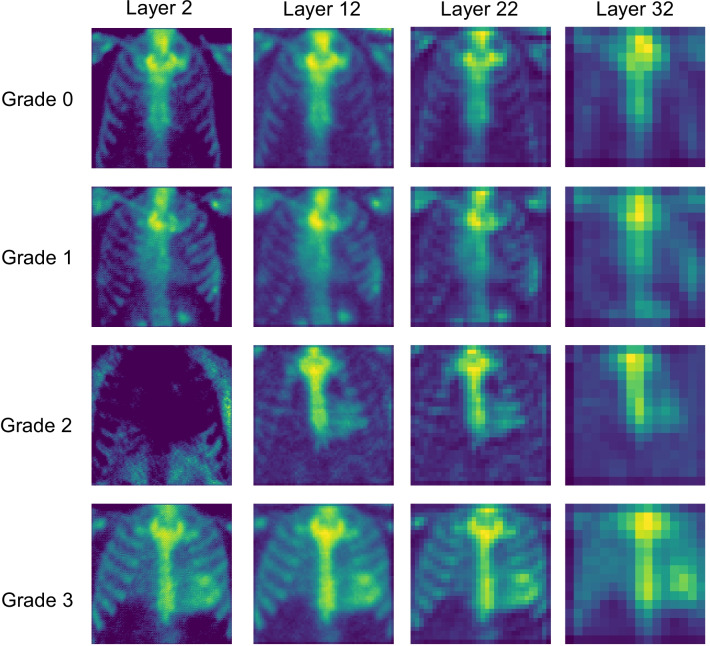
Maximum activation maps for layers 2, 12, 22 and 32 of the Residual model for input images representing different grades of cardiac uptake. Activation maps demonstrate that the convolutional neural network model finds abnormal myocardial signal in patients with cardiac uptake suggestive for ATTR similarly to physician and not extracardiac features similarly to standard clinical reading by physician

The maximum activation maps for layers 2, 10, 17 and 24 of the Linear model are shown in Fig. 4, and for layers 2, 12, 22 and 32 of the Residual model in Fig. 5. The figures demonstrate how both models emphasized characteristic image features for different classes. For patients with Perugini grades 0 and 1, the activation maps show the highest weights around the sternum, shoulders and ribs; and for patients with grades 2 and 3, in the sternum and heart.

## Discussion

In the current study, we classified different grades of cardiac uptake on bone scintigraphy images using automated convolutional neural networks. We found that two custom-made CNN models performed better in this classification task than four state-of-the-art models implemented in the Keras library. It is noteworthy that the imaging data were heterogeneous, since the scintigraphy images were collected with multiple different scanners during a time span of nine years and included both planar and whole-body images. The dataset was imbalanced since only 4% of patients had cardiac uptake positive for ATTR similarly to real-world clinical practice. Yet, our CNN models could accurately detect and classify different grades of cardiac uptake.

Similarly to clinical practice, patients with cardiac uptake around the visual cut-off value (grade 1 vs. 2) were the most difficult to discriminate. In contrast, absence of cardiac uptake or high-grade uptake (grade 3) were classified with a high precision using both of our CNN models. In the diagnosis of ATTR (> grade 2), the Linear and Residual models performed better than, or equally well as, the state-of-the-art models. Although VGG16 performed quite well in the current dataset, it was the most computationally heavy of the tested models, consisting of 138 million trainable parameters and requiring 528 MB of memory. In comparison, the Linear and Residual models both included only 1 million parameters, making them much more efficient.

### Previous studies in brief

While deep learning has previously been used to diagnose cardiac amyloidosis from MRI and PET images [11, 12], cardiac uptake from bone scintigraphy images has not been previously studied. In previous studies, CNNs have performed well in detecting metastases in whole-body bone scintigraphy images. Papandrianos and colleagues reported a 96% sensitivity for detection of prostate cancer metastases with a simple CNN model [7]. However, they manually removed artifacts (e.g., injection site) from the images before applying the model and discarded patients with degenerative lesions from their dataset. In a later article from the same group [8], a more complicated CNN architecture classified bone scintigraphy images into normal, metastatic lesion and degenerative lesion categories with 97% accuracy. Zhao and colleagues presented similar results for detection of bone metastases from different cancer types [5]. Their CNN approach yielded 0.96–0.99 AUCs depending on the primary cancer type, and the data included patients with degenerative lesions as well. Recently, good results in bone metastasis analysis have also been reported in other studies [6, 9, 10].

While the previous studies regarding the use of deep learning in nuclear medicine are promising, they cannot be directly compared to the current study. Firstly, the uptake of bone metastases is typically higher than myocardial signal in ATTR, which makes the metastases more easily detectable. Secondly, metastatic lesions and image artifacts have not been removed from our dataset. Thirdly, distinguishing between different visual grades of cardiac uptake is subjective. However, our primary clinical aim is to aid screening of incidental cardiac uptake and not to substitute visual analysis with machine learning methods. Positive findings would be confirmed by a physician or by further multimodality imaging, as necessary [2].

### CNN layer visualization

The visualization of maximal activation maps for different CNN layers revealed that both the Linear and Residual models were highlighting similar features in the images. For negative patients (grade 0 or 1), the highest weights were located in bones, and for ATTR patients, the highest weights were located in the heart. Thus, CNNs are focusing, like reading physicians, on clinically relevant features in the images and have appropriately learned these features from the training data. This finding is important regarding to the ethical principles of deep learning in medicine as it provides transparency and visibility to the decisions made by the CNNs [21]. On the other hand, it seemed that the CNN models did not find any additional relevant features which would have been unnoticed by the physician; it remains to be investigated in further studies whether such incidental findings emerge when the models are trained with larger datasets.

### Clinical significance of the results

Bone scintigraphy is a common imaging procedure, and one of its main indications is prostate cancer which is frequent in elderly males. This results in overlap between a population with indication to bone scintigraphy and a population where the incidental ATTR is the most prevalent [22]. Scintigraphy is positive in the early stage of an amyloid process, even before echocardiography [23] Therefore, automated CNN analysis might result in earlier diagnosis of ATTR by detecting cases missed by clinical reading or by large scale screening of bone scintigraphy databases. Patients with subclinical cardiac uptake suggestive for early amyloid process represent an interesting target population for further studies. Automated analysis might also decrease the interobserver variation of visual reading in clinicians with less exposure to amyloid imaging.

For patients with suspected cardiac amyloidosis, the diagnostic yield of our automated analysis would be low, as they will undergo SPECT/CT in any case and the images will be read by a physician with expertise in nuclear cardiology. However, the value of automated screening of bone scans lies in the warning of less experienced technicians during the quality check of whole-body images. In case of a positive ATTR finding, additional cross-sectional imaging can be acquired to confirm the diagnosis. This will reduce the need for repeated nuclear imaging and unnecessary radiation exposure. Furthermore, although the visual analysis of planar images has been shown to be accurate in previous studies [23, 24], ESC currently recommends the use of SPECT or SPECT/CT for diagnosis of ATTR [1]. Thus, possible treatment decisions would be made based on cross-sectional images and further multimodality imaging according to ESC position statement [1].

### Limitations and future work

The CNN architectures in the current study were fairly simple: the Linear model included mainly consecutive convolutional layers and pooling layers, and the Residual model had additional skip connections between every other layer. More complicated architectures could be designed to improve accuracy. However, it seemed that very deep CNNs, such as ResNet50, did not perform as well as our models, probably due to overfitting. Thus, merely increasing the depth of the CNN is likely not beneficial if there is no simultaneous increase in training data.

Another limitation in our study was the low number of patients with cardiac uptake suggestive for ATTR. Although the size of the dataset was in principle sufficient for deep learning (1334 images), the number of positive patients was low and unbalanced. Therefore, we used weighted probabilities for different classes to compensate for the skewed study population. More data from patients with cross-sectional imaging for comparison would be necessary to improve the classification. CNN analysis of whole-body images might have resulted in better differentiation of grade 2 versus 3 cardiac uptake. However, we chose the current heart-focused approach requiring less computational power. Of note, our study population was selected and cannot be used to study the prevalence of cardiac uptake. Visual analysis of cardiac uptake is subjective, and we chose to emphasize sensitive reading of images as our machine learning algorithm would be applied to oncologic population with very low pretest probability of ATTR. Initial screening with high sensitivity would then be supplemented by cross-sectional imaging with high specificity in the same imaging session. Final diagnosis would be done by the reading physician. HMDP is less validated for amyloid imaging compared to PYP or DPD but it is commonly used in bone scintigraphy imaging. Therefore, our study represents the real-world target-group for screening of ATTR.

One way to improve the detection of myocardial signal would be focusing the analysis more strictly to the cardiac region by cropping or smaller image matrix centered in the heart. This would require localization of the heart before the CNN analysis, either manually or with an automated feature detection algorithm. It is also possible to implement a CNN-based cardiac segmentation workflow and classify each pixel to a different category, e.g., normal or abnormal signal. However, it would require the ground truth labels for each pixel. Thus, a physician would have to manually segment all images before the CNN training.

In the future, the proposed CNNs could be implemented into an automated image analysis workflow which could assist the physicians in detection of incidental ATTR on bone scintigraphy. A possible analysis pipeline could be structured as follows: 1) whole-body bone scintigraphy images are sent directly from the scanner to an analysis server, 2) the images are automatically preprocessed and classified, 3) if the classification result suggests cardiac uptake, an alert message is sent to either the technician or physician, and 4) the physician can further inspect the whole-body images and decide whether additional cross-sectional imaging is needed and refer the patient to a cardiologist.

## Conclusions

Convolutional neural networks can accurately detect and classify different grades or cardiac uptake. The Linear and Residual CNN models performed better than the state-of-the-art models. In addition, the maximum activation maps of these models showed that the CNN models are focused on cardiac region on patients with cardiac uptake suggestive for ATTR. These models could be used for automated screening of ATTR from bone scintigraphy images.

## Data Availability

The data analyzed during the current study are available from the corresponding author on reasonable request.

## Abbreviations

ATTR: Transthyretin amyloidosis
AUC: Area under the curve
CNN: Convolutional neural network
DPD: Diphosphono-1,2-propanodicarboxylicacid
HMDP: Hydroxymethylene diphosphonate
PYP: Pyrophosphate
ROC: Receiver operating characteristic
SPECT: Single-photon emission computed tomography
